# Severity of low pre-pregnancy body mass index and perinatal outcomes: the Japan Environment and Children’s Study

**DOI:** 10.1186/s12884-022-04418-3

**Published:** 2022-02-11

**Authors:** Kentaro Nakanishi, Yasuaki Saijo, Eiji Yoshioka, Yukihiro Sato, Yasuhito Kato, Ken Nagaya, Satoru Takahashi, Yoshiya Ito, Sumitaka Kobayashi, Chihiro Miyashita, Atsuko Ikeda-Araki, Reiko Kishi, the Japan Environment and Children’s Study (JECS) Group, Michihiro Kamijima, Shin Yamazaki, Yukihiro Ohya, Nobuo Yaegashi, Koichi Hashimoto, Chisato Mori, Shuichi Ito, Zentaro Yamagata, Hidekuni Inadera, Takeo Nakayama, Hiroyasu Iso, Masayuki Shima, Youichi Kurozawa, Narufumi Suganuma, Koichi Kusuhara, Takahiko Katoh

**Affiliations:** 1 Department of Obstetrics and Gynecology, Asahikawa Medical University, 1-1-1, Midorigaoka higashi2-jo, Asahikawa, Hokkaido 078-8510 Japan; 2 Division of Public Health and Epidemiology, Department of Social Medicine, Asahikawa Medical University, 1-1-1, Midorigaoka higashi2-jo, Asahikawa, Hokkaido 078-8510 Japan; 3 Division of Neonatology, Perinatal Medical Center, Asahikawa Medical University Hospital, 1-1-1, Midorigaoka higashi2-jo, Asahikawa, Hokkaido 078-8510 Japan; 4 Department of Pediatrics, Asahikawa Medical University, 1-1-1, Midorigaoka higashi2-jo, Asahikawa, Hokkaido 078-8510 Japan; 5 Faculty of Nursing, Japanese Red Cross Hokkaido College of Nursing, 664-1, Akebono-cho, Kitami, Hokkaido 090-0011 Japan; 6 Center for Environmental and Health Sciences, Hokkaido University, Kita12-jo, Nishi7-chome, Kita-ku, Sapporo, Hokkaido 060-0812 Japan; 7 Faculty of Health Sciences, Hokkaido University, Kita12-jo, Nishi5-chome, Kita-ku, Sapporo, Hokkaido 060-0812 Japan

**Keywords:** Adverse perinatal outcomes, Low birth weight, Maternal body mass index, Preterm birth, Small-for-gestational age

## Abstract

**Background:**

The extremes of maternal pre-pregnancy body mass index (BMI) are known to be risk factors associated with obstetric and adverse perinatal outcomes. Among Japanese women aged 20 years or older, the prevalence of underweight (BMI < 18.5 kg/m^2^) was 11.5% in 2019. Maternal thinness is a health problem caused by the desire to become slim. This study aimed to investigate the association between the severity of maternal low pre-pregnancy BMI and adverse perinatal outcomes, including preterm birth (PTB), low birth weight (LBW), and small-for-gestational age (SGA).

**Methods:**

We conducted a prospective cohort study using data from the Japan Environment and Children’s Study, which recruited pregnant individuals between 2011 and 2014. Pre-pregnancy BMI was categorized as severe-moderate underweight (BMI < 16.9 kg/m^2^), mild underweight (BMI, 17.0–18.4 kg/m^2^), low-normal weight (BMI, 18.5–19.9 kg/m^2^), high-normal weight (BMI, 20.0–22.9 kg/m^2^), overweight (BMI, 23.0–24.9 kg/m^2^), and obese (BMI ≥ 25.0 kg/m^2^). The high-normal weight group was used as the reference for statistical analyses. Adjusted logistic regression was performed to evaluate the association between pre-pregnancy BMI and PTB, LBW, and SGA.

**Results:**

Of 92,260 singleton pregnant individuals, the prevalence was 2.7% for severe-moderate underweight, 12.9% for mild underweight, and 24.5% for low-normal weight. The prevalence of adverse outcomes was 4.6% for PTB, 8.1% for LBW, and 7.6% for SGA. The adjusted odds ratios (aORs) for PTB were 1.72 (95% confidence interval [CI], 1.46–2.03) for severe-moderate underweight and 1.26 (95% CI, 1.14–1.39) for mild underweight. The aORs of LBW were 2.55 (95% CI, 2.27–2.86) for severe-moderate underweight, 1.64 (95% CI, 1.53–1.76) for mild underweight, and 1.23 (95% CI, 1.16–1.31) for low-normal weight. The aORs of SGA were 2.53 (95% CI, 2.25–2.84) for severe-moderate underweight, 1.66 (95% CI, 1.55–1.79) for mild underweight, and 1.29 (95% CI, 1.21–1.38) for low-normal weight.

**Conclusions:**

A dose-response relationship was found between the severity of low pre-pregnancy BMI and PTB, LBW, and SGA. Even low-normal BMI (18.5–19.9 kg/m^2^) increased the risk of LBW and SGA. This study provides useful information for pre-conception counseling in lean individuals.

**Supplementary Information:**

The online version contains supplementary material available at 10.1186/s12884-022-04418-3.

## Background

The extremes of maternal pre-pregnancy body mass index (BMI) are known to be risk factors associated with obstetric and adverse perinatal outcomes [1–7]. Higher maternal pre-pregnancy BMI increases the risk of hypertensive disorders during pregnancy, gestational diabetes, preterm birth (PTB), fetal death, stillbirth, large-for-gestational age, and macrosomia [2–7]. A lower maternal pre-pregnancy BMI increases the risk of PTB, low birth weight (LBW), and small-for-gestational age (SGA) [1, 3–11].

In Japan, maternal underweight is a common health problem because many individuals in the reproductive-age strongly desire to be slim and work towards it [12]. Among Japanese women aged 20 years or older, the prevalence of underweight individuals (< 18.5 kg/m^2^) was 11.5% in the 2019 National Health and Nutrition Survey of Japan [13]. In particular, this figure among Japanese women in their 20’s was 20.7%. Indeed, the frequency of LBW has been increasing in Japan despite the rate of PTB being as low as 5.6% in 2019 [14]. Several studies reported that low maternal pre-pregnancy BMI was significantly associated with adverse perinatal outcomes, such as PTB, LBW, and SGA [1, 4, 5, 7–11, 15]. However, to our knowledge, no studies on the severity of low pre-pregnancy BMI have been conducted in East Asian countries including Japan where pregnancy-aged individuals have a lower BMI compared to other regions. Further, no prospective cohort studies examining the adverse obstetric and perinatal outcomes associated with severity of pre-pregnancy underweight were found. Although two large cohort studies in Japan reported the association between pre-pregnancy underweight and PTB and SGA, the severity of maternal pre-pregnancy underweight was not evaluated [3, 16].

The definition of BMI in Asia differs from that in the United States and European countries because the prevalence of underweight pregnant individuals in Asia is higher than that in other countries [17]. The appropriate BMI categories recommended by the World Health Organization (WHO) for Asian populations are as follows: less than 18.5 kg/m^2^, underweight; 18.5–23 kg/m^2^, increasing but acceptable risk; 23–27.5 kg/m^2^, increased risk; and ≥ 27.5 kg/m^2^, high risk [17]. The WHO also states that countries should use all BMI categories, with a view to facilitate international comparisons as follows: the trigger points are 18.5, 20, 23, 25, 27.5, 30, and 32.5 kg/m^2^ [17]. Thus, BMI needs to be classified according to this WHO categorization to conduct the study on pre-pregnancy BMI in Asian populations.

Additionally, few studies have quantified the association between maternal pre-pregnancy BMI and adverse perinatal outcomes, using intuitive methods such as the restricted cubic spline (RCS). The RCS has the advantages of being able to characterize a dose-response association between a continuous exposure and an outcome, and check the assumption of linearity of the association visually and statistically [18].

The aim of the present study was to investigate the association between the severity of maternal low pre-pregnancy BMI and gestational age at delivery and neonatal birth weight using a nationwide, prospective cohort study of approximately 100,000 mothers and neonates. In addition, we used the RCS to analyze the association between maternal pre-pregnancy BMI and adverse perinatal outcomes, including PTB, LBW, and SGA.

## Methods

### Study design and participants

We conducted a prospective cohort study using the dataset jecs-ta-20190930, which was released in October 2019, from the Japan Environment and Children’s Study (JECS). The JECS is an ongoing nationwide birth cohort study, the details of which have already been published [19, 20]. The JECS mainly aimed to investigate the association between environmental factors and children’s health and development by recruiting pregnant individuals from 15 Regional Centers between January 2011 and March 2014. However, in 2013, when recruitment was largely stabilized, approximately only 45% of children could be assessed [20].

**Fig. 1:**
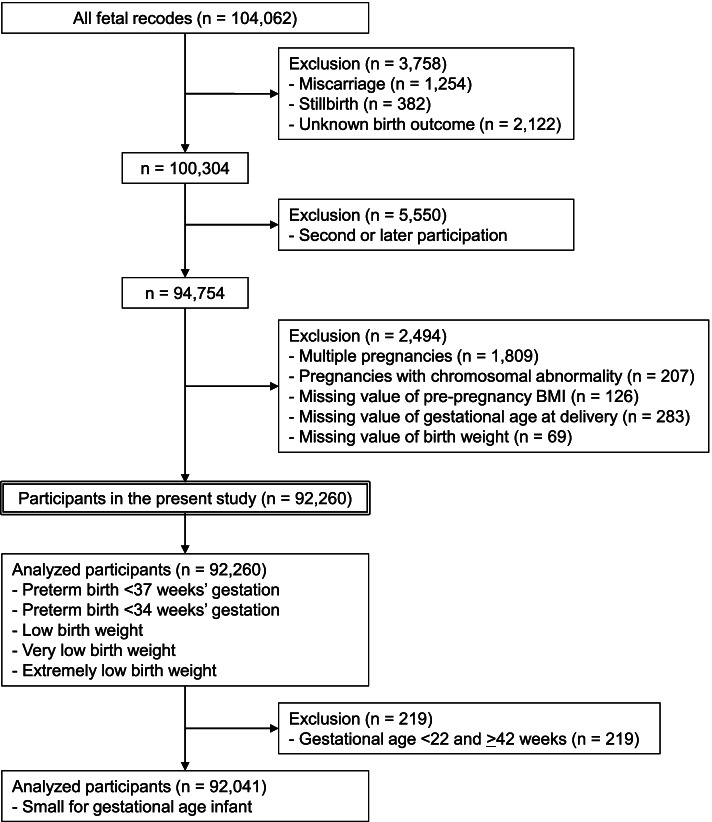
Flow diagram of the study participants

Between January 2011 and March 2014, 104,062 fetal recodes were enrolled in the JECS. After excluding miscarriage (*n* = 1254), stillbirth (*n* = 382), and unknown birth outcome (*n* = 2122), the present study included 100,304 live births. Then, after excluding pregnancies in the same participants, the study involved 94,754 participants. Finally, after excluding multiple pregnancies (*n* = 1809), and pregnancies with chromosomal abnormality (*n* = 207), missing value of maternal pre-pregnancy BMI (*n* = 126), missing value of gestational age at delivery (*n* = 283), and missing value of neonatal birth weight (*n* = 69), the number of study participants was 92,260 singleton, pregnant individuals (Fig. 1). The number of participants in the SGA analyses decreased to 92,041 because we excluded 219 deliveries that were performed at a gestational age of < 22 weeks or > 41 weeks (Fig. 1).

### Ethics

The study protocol was reviewed and approved by the Institutional Review Board on Epidemiological Studies of the Ministry of the Environment and by the Ethics Committees of all participating institutions (No. 100910001) [19]. This study was conducted in accordance with the Declaration of Helsinki. All participants in the JECS provided written informed consent.

### Maternal and neonatal baseline information

Maternal baseline information, including maternal educational background, smoking status, and alcohol consumption was obtained from self-administered questionnaires provided to the enrolled pregnant individuals during the second/third trimester. The following information was also collected from their medical record transcripts: parity, assisted reproductive technology, multiple births, maternal history of preterm birth less than 37 weeks of gestation, medical histories of hypertension, diabetes mellitus, autoimmune disease, thyroid disease such as hyperthyroidism and hypothyroidism, neonate’s date of birth, and gestational period. Although maternal height and pre-pregnancy weight were, in principle, obtained from medical record transcripts, in instances where the above information (maternal height and pre-pregnancy weight) was missing in medical record transcripts, the values were obtained from self-reports of participants. The mother’s age at the time of childbirth was calculated from the mother’s and the neonate’s birth dates.

Pre-pregnancy BMI was defined as the maternal pre-pregnancy weight divided by the square of maternal height obtained from medical record transcripts or self-reports. Pregnant individuals were categorized based on their pre-pregnancy BMI as follows: severe-moderate underweight (BMI < 16.9 kg/m^2^), mild underweight (BMI, 17.0–18.4 kg/m^2^), low-normal weight (BMI, 18.5–19.9 kg/m^2^), high-normal weight (BMI, 20.0–22.9 kg/m^2^), overweight (BMI, 23.0–24.9 kg/m^2^), and obese (BMI > 25.0 kg/m^2^), with reference to the WHO’s classification for BMI [12, 21]. The high-normal pre-pregnancy BMI group was the reference group for statistical analyses.

Parity was categorized as 0, 1, 2, or higher. Maternal smoking status was defined as follows: never or previously did, but quit before recognizing current pregnancy; previously did, but quit after identifying current pregnancy, and; yes, I still smoke. Maternal alcohol consumption was defined as follows: never or previously did, but quit before identifying current pregnancy; previously did, but quit after identifying current pregnancy, and; yes, I drink. The highest level of education of the mother was defined as follows: junior high school, high school, technical junior college, technical/vocational college or associate degree, bachelor’s degree, or Graduate degree (Master’s/Doctor’). Medical information of the mother and neonate, including maternal age at delivery, gestational age at delivery, neonatal birth weight, and neonatal sex, was transcribed from the medical record transcripts at delivery.

### Outcomes

The main outcomes of the present study were the incidence of adverse perinatal outcomes, including PTB, very preterm birth (VPTB), LBW, very low birth weight (VLBW), extremely low birth weight (ELBW), and SGA. PTB and VPTB were defined as the gestational age of less than 37 weeks and 34 weeks at delivery, respectively. LBW, VLBW, and ELBW were defined as neonatal birth weights of less than 2500 g, 1500 g, and 1000 g, respectively. SGA was defined as a birth weight below the 10th percentile, accounting for infant sex, parity, and gestational age according to the Japan Pediatric Society [22], and the percentiles were calculated using Excel-based clinical tools for growth evaluation of children distributed by the Japanese Society for Pediatric Endocrinology [23].

### Statistical analysis

The adjusted odds ratio (OR) and 95% confidence interval (CI) for adverse perinatal outcomes were calculated by maternal pre-pregnancy BMI compared to the reference group (BMI, 20.0–22.9 kg/m^2^, high-normal weight) using a multivariable logistic regression model, adjusted for maternal age at delivery, parity (except for SGA analysis), assisted reproductive technology, maternal smoking status, maternal alcohol consumption, maternal educational background, maternal history of preterm birth, and the medical history of hypertension, diabetes mellitus, autoimmune disease, and thyroid disease such as hyperthyroidism and hypothyroidism.

The RCSs were used to detect a dose-response relationship between pre-pregnancy BMI and each adjusted OR of the adverse perinatal outcomes. The RCS models the association between predictor and outcome using cubic polynomials and linear terms [24]. The RCS requires the placement of a number of knots along the predictor value range. Usually, three to five knots are sufficient to allow for complex associations between predictors and outcomes; the present study was analyzed with five knots placed at the 5th, 27.5th, 50th, 72.5th, and 95th percentiles of the BMI distribution.

Because the dataset had some missing values, based on the assumption of missing at random, the k-nearest neighbor (kNN) imputation method of the R package “VIM” (ver. 4.1.2; R Foundation for Statistical Computing) was used [25], introducing all outcomes and adjusted variables, along with gestational weeks. The kNN imputation method is a widely accepted single imputation method, and its validity has been confirmed [26]. The kNN imputation was appropriate for the RCS analysis. In addition, we performed a sensitivity analysis using the complete dataset and excluded all missing values. The results of the sensitivity analysis are shown in the Supplemental Table. Statistical significance was set at a two-tailed *P* value of < 0.05. All analyses, except k-nearest neighbor imputation, were performed using STATA version 16.1, for Windows (Stata Corporation, College Station, TX, USA).

## Results

In the present study, 99.8% (92,089/92,260) of the maternal pre-pregnancy weights were obtained from medical record transcripts and 0.2% (171/92,260) from self-reported data. Of the participants who had both sets of data, 65.4% reported the same weight in both sets. The high-normal weight group (BMI, 20.0–22.9 kg/m^2^), which was the reference group, had the highest prevalence (38.5%) among all pre-pregnancy BMI categories (Table 1). The prevalence of low maternal pre-pregnancy BMI was as follows: 15.7% (14,448/92,260) for underweight (BMI < 18.4 kg/m^2^) and 24.5% (22,583/92,260) for low-normal weight (BMI, 18.5–19.9 kg/m^2^). Of pre-pregnant individuals who were underweight, the prevalence was 17.4% (2515/14,448) for severe-moderate underweight (BMI < 16.9 kg/m^2^) and 82.6% (11,933/14,448) for mild underweight (BMI, 17.0–18.4 kg/m^2^).

**Table 1:** Maternal and neonatal characteristics (*n* = 92,260)

Characteristics	Values^a^
Maternal age (years)	
< 24	9252 (10.0)
25–29	25, 374 (27.5)
30–34	32,543 (35.3)
35–39	20,838 (22.6)
> 40	4247 (4.6)
Missing	6 (0.01)
Parity	
0	38,291 (41.5)
1	33,694 (36.5)
> 2	17,985 (19.5)
Missing	2290 (2.5)
Assisted reproductive technology	
No	89,068 (96.5)
Yes	2761 (3.0)
Missing	431 (0.5)
Pre-pregnancy BMI	
< 16.9, Severe to moderate underweight	2515 (2.7)
17.0–18.4, Mild underweight	11,933 (12.9)
18.5–19.9, Low-normal weight	22,583 (24.5)
20.0–22.9, High-normal weight	35,537 (38.5)
23.0–24.9, Overweight	9658 (10.5)
> 25.0, Obese	10,034 (10.9)
Smoking during pregnancy	
No	73,263 (79.4)
Quit after pregnancy	12,636 (13.7)
Yes	4103 (4.4)
Missing	2258 (2.5)
Drinking during pregnancy	
No	44,991 (48.8)
Quit after pregnancy	42,529 (46.1)
Yes	2497 (2.7)
Missing	2243 (2.4)
Maternal educational background	
Junior high school	4326 (4.7)
High school	28,318 (30.7)
Technical junior college or technical/vocational college	37,991 (41.2)
University or above	19,601 (21.2)
Missing	2024 (2.2)
History of preterm birth	
No	86,753 (94.0)
Yes	2853 (3.1)
Missing	2654 (2.9)
Hypertension	
No	89,408 (96.9)
Yes	1162 (1.3)
Missing	1690 (1.8)
Diabetes mellitus	
No	89,571 (97.1)
Yes	995 (1.1)
Missing	1694 (1.8)
Autoimmune disease	
No	90,377 (98.0)
Yes	183 (0.2)
Missing	1700 (1.8)
Thyroid disease	
No	89,344 (96.8)
Yes	1224 (1.3)
Missing	1692 (1.8)
Gestational age at delivery (weeks)	39 (22–43)
Birth weight (grams)	3028 (312–5214)
Neonatal sex	
Male	47,329 (51.3)
Female	44,924 (48.7)
Missing	7 (0.01)
Preterm birth (< 37 weeks’ gestation)	4284 (4.6)
Very preterm birth (< 34 weeks’ gestation)	907 (1.0)
Low birth weight (< 2500 g)	7514 (8.1)
Very low birth weight (< 1500 g)	528 (0.6)
Extremely low birth weight (< 1000 g)	228 (0.3)
Small-for-gestational age	
No	85,029 (92.2)
Yes	7012 (7.6)
Missing	219 (0.2)

^a^Values are presented as n (%) or median (range)

In the total study population, the prevalence of PTB was 4.6% and that of VPTB was 1.0% (Table 1). Regarding neonatal birth weight, the prevalence of LBW was 8.1%, of VLBW was 0.6%, of ELBW was 0.3%, and of SGA was 7.6% (Table 1).

Low maternal pre-pregnancy BMI was significantly associated with PTB, LBW, and SGA (Table 2). Regarding low pre-pregnancy BMI, the aORs of PTB were 1.72 (95% CI, 1.46–2.03; *P* < 0.001) for severe-moderate underweight and 1.26 (95% CI, 1.14–1.39; *P* < 0.001) for mild underweight. The aORs of LBW were 2.55 (95% CI, 2.27–2.86; *P* < 0.001) for severe-moderate underweight, 1.64 (95% CI, 1.53–1.76; *P* < 0.001) for mild underweight, and 1.23 (95% CI, 1.16–1.31, *P* < 0.001) for low-normal weight. The aORs of SGA were 2.53 (95% CI, 2.25–2.84; *P* < 0.001) for severe-moderate underweight, 1.66 (95% CI, 1.55–1.79; *P* < 0.001) for mild underweight, and 1.29 (95% CI, 1.21–1.38; *P* < 0.001) for low-normal weight (Table 2). In the complete dataset, maternal low pre-pregnancy BMI was also significantly associated with PTB, LBW, and SGA (Supplemental Table 1).

**Table 2:** Odds ratios of maternal pre-pregnancy body mass index for adverse perinatal outcomes after imputation (*n* = 92,260)

Outcomes	Pre-pregnancy body mass index (kg/m^2^)					
	< 16.9	17.0–18.4	18.5–19.9	20.0–22.9	23.0–24.9	25.0 <
**PTB**						
N (%)	171 (3.99)	604 (14.10)	960 (22.41)	1477 (34.48)	449 (10.48)	623 (14.54)
Crude OR (95% CI)	1.68 (1.43–1.98)	1.23 (1.12–1.35)	1.02 (0.94–1.11)	reference	1.12 (1.01–1.25)	1.53 (1.39–1.68)
Adjusted OR (95% CI) ^a^	**1.72 (1.46–2.03)**	**1.26 (1.14–1.39)**	1.05 (0.96–1.14)	reference	1.07 (0.96–1.20)	**1.36 (1.23–1.51)**
**VPTB**						
N (%)	29 (3.20)	112 (12.35)	181 (19.96)	304 (33.52)	112 (12.35)	169 (18.63)
Crude OR (95% CI)	1.35 (0.92–1.98)	1.10 (0.88–1.37)	0.94 (0.78–1.13)	reference	1.36 (1.09–1.69)	1.99 (1.64–2.40)
Adjusted OR (95% CI) ^a^	1.36 (0.92–2.00)	1.12 (0.90–1.39)	0.96 (0.79–1.15)	reference	**1.28 (1.03–1.59)**	**1.72 (1.42–2.09)**
**LBW**						
N (%)	404 (5.38)	1297 (17.26)	1896 (25.23)	2499 (33.26)	678 (9.02)	740 (9.85)
Crude OR (95% CI)	2.53 (2.26–2.84)	1.61 (1.50–1.73)	1.21 (1.14–1.29)	reference	1.00 (0.91–1.09)	1.05 (0.97–1.15)
Adjusted OR (95% CI) ^a^	**2.55 (2.27–2.86)**	**1.64 (1.53–1.76)**	**1.23 (1.16–1.31)**	reference	0.96 (0.88–1.05)	0.96 (0.88–1.04)
**VLBW**						
N (%)	17 (3.22)	69 (13.07)	104 (19.70)	168 (31.82)	65 (12.31)	105 (19.89)
Crude OR (95% CI)	1.43 (0.87–2.36)	1.22 (0.92–1.62)	0.97 (0.76–1.24)	reference	1.43 (1.07–1.90)	2.23 (1.74–2.84)
Adjusted OR (95% CI) ^a^	1.45 (0.88–2.40)	1.25 (0.5–1.66)	1.00 (0.78–1.27)	reference	1.33 (1.00–1.78)	**1.82 (1.41–2.35)**
**ELBW**						
N (%)	5 (2.19)	28 (12.28)	45 (19.74)	83 (36.40)	23 (10.09)	44 (19.30)
Crude OR (95% CI)	0.85 (0.34–2.10)	1.00 (0.65–1.54)	0.85 (0.59–1.23)	reference	1.02 (0.64–1.62)	1.88 (1.30–2.71)
Adjusted OR (95% CI) ^a^	0.84 (0.34–2.07)	1.03 (0.67–1.58)	0.87 (0.61–1.25)	reference	0.95 (0.60–1.51)	**1.56 (1.07–2.27)**
**SGA**^**b**^						
N (%)	381 (5.43)	1245 (17.76)	1871 (26.68)	2349 (33.50)	605 (8.63)	561 (8.00)
Crude OR (95% CI)	2.52 (2.24–2.83)	1.64 (1.53–1.77)	1.27 (1.20–1.36)	reference	0.94 (0.86–1.04)	0.84 (0.76–0.92)
Adjusted OR (95% CI) ^a^	**2.53 (2.25–2.84)**	**1.66 (1.55–1.79)**	**1.29 (1.21–1.38)**	reference	0.92 (0.84–1.01)	**0.78 (0.71–0.86)**

The adjusted odds ratios with statistical significance are presented in bold

^a^The odds ratio compared to that of infants of mothers with the high-normal pre-pregnancy body mass index (20.0–22.9 kg/m^2^), adjusted for maternal age at delivery, parity (except for SGA analysis), assisted reproductive technology, maternal smoking status, maternal alcohol consumption, maternal educational background, history of preterm birth, medical history of hypertension, diabetes mellitus, autoimmune disease, and thyroid disease

^b^The total number of participants was 92,041

For VPTB, VLBW, and ELBW, significant differences were identified in maternal pre-pregnancy obese group compared with the reference group (Table 2). However, no significant difference was found between the low pre-pregnancy BMI groups.

**Fig. 2:**
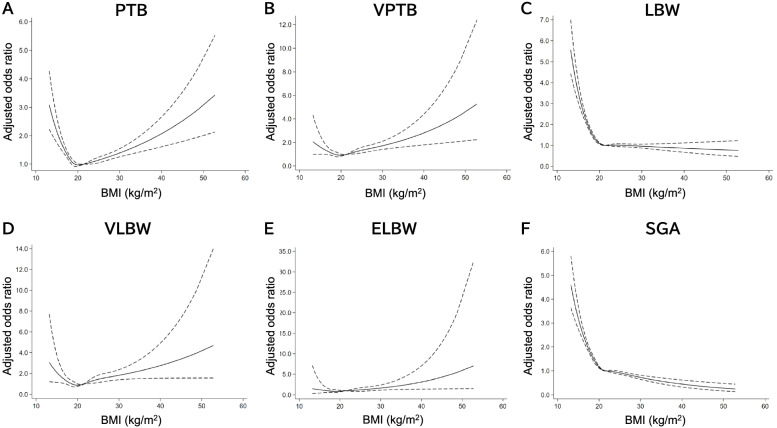
Maternal pre-pregnancy BMI and the risk of adverse perinatal outcomes using restricted cubic spline. Values are adjusted odds ratio and 95% confidence interval compared with the referent group (high-normal weight, 20.0–22.4 kg/m^2^), adjusted for maternal age at delivery, parity (except for small-for-gestational age [SGA] analysis), assisted reproductive technology, maternal smoking status, maternal alcohol consumption, maternal educational background, history of preterm birth, and the medical history of hypertension, diabetes mellitus, autoimmune disease, and thyroid disease. The adjusted odds ratios are shown as solid lines. The 95% confidence interval is indicated by dotted lines. **A** PTB: preterm delivery before 37 weeks of gestation. **B** VPTB: preterm delivery before 34 weeks of gestation. **C** LBW: low birth weight **D** VLBW: very low birth weight. **E** ELBW: extremely low birth weight. **F** SGA: small-for-gestational age. BMI, body mass index

Figure 2 demonstrates the non-linear association between maternal pre-pregnancy BMI and adverse perinatal outcomes using 5 knots RCS with adjustment for confounders. The dose-response relationship between pre-pregnancy BMI and PTB, LBW, and SGA was observed only in the low BMI range (Fig. 2A, C, F). The RCS also showed a U-shaped relationship between maternal pre-pregnancy BMI and VPTB and VLBW (Fig. 2B, D).

## Discussion

We further divided low pre-pregnancy BMI into two levels (< 16.9 kg/m^2^, and 17.0–18.4 kg/m^2^) and analyzed the association between the two levels of low BMI and low-normal BMI (18.5–19.9 kg/m^2^) and adverse perinatal outcomes. To our knowledge, this is the first study to evaluate the association between the severity of maternal low pre-pregnancy BMI and adverse perinatal outcomes in Japanese women, in whom a high prevalence of thinness can be observed. The dose-response relationship between the severity of low pre-pregnancy BMI and PTB, LBW, and SGA was found only in the low BMI range. In addition, the present study focused on adverse perinatal outcomes, such as earlier PTB and LBW. However, the association between low maternal pre-pregnancy BMI and VPTB, VLBW, and ELBW was not statistically significant.

The association between underweight pre-pregnant individuals and PTB has been well studied previously [1, 5, 9–11, 15]. However, only a few studies have been reported on the association between the severity of low pre-pregnancy BMI and PTB [1, 9–11]. There are four studies that demonstrated that the severe and moderate pre-pregnant underweight groups significantly increased the risk of PTB compared with normal weighing groups [1, 9–11]. Except for the study by Lynch et al. [1], the mildly underweight pre-pregnant group was associated with increased risk of PTB in three studies [9–11]. Moreover, a meta-analysis on the association between pre-pregnancy BMI and pregnancy complications also demonstrated that underweight pre-pregnant individuals were at higher risk of experiencing PTB [5]. In the present study, the pre-pregnancy underweight group was at a significantly higher risk of PTB compared with the high-normal weight group. Regarding the severity of low pre-pregnancy BMI, our results show that the lower the pre-pregnancy BMI, the greater the risk of PTB. Therefore, our results are consistent with those of previous studies.

In the present study, underweight pre-pregnant individuals were not significantly associated with an increased risk of PTB before 34 weeks of gestation compared with the high-normal weight group. In the study by Shaw et al. [15], there was no significant association between maternal pre-pregnancy underweight and spontaneous PTB before 32 weeks of gestation. However, Salihu et al. [9] and Ratnasiri et al. [6] reported that maternal pre-pregnancy underweight was significantly associated with an increased risk of PTB before 33 and 32 weeks of gestation, respectively. Interestingly, Ratnasiri et al. [6] reported that the aOR was lower for PTB before 32 weeks of gestation than for PTB before 37 weeks of gestation. This finding, stating that low pre-pregnancy BMI had a smaller impact on early PTB, was similar to our results. In the present study, earlier PTB was strongly associated with higher pre-pregnancy BMI than lower pre-pregnancy BMI. However, we found that the risk of VPTB tended to increase with lower pre-pregnancy BMI because the RCS showed a U-shaped relationship between low pre-pregnancy BMI and VPTB.

The association between maternal pre-pregnancy underweight and neonatal birth weight has been reported in many studies [3, 5, 6, 8, 27–31]; however, only one study by Salmon et al. [11] investigated the association between the severity of low pre-pregnancy BMI and neonatal birth weight. They reported that severe-moderate and mild underweight maternal pre-pregnancy BMIs were significantly associated with an increased risk of intrauterine growth restriction, LBW, and VLBW with a dose-response relationship. They reported that the value of aOR was lower for birth weights less than 1500 g than for those weighing less than 2500 g, which is similar to the results of our study. In addition, the meta-analysis by Santos et al. [5] demonstrated that a lower pre-pregnancy BMI was associated with an increased risk of SGA. The present study demonstrated that the risk of LBW and SGA was significantly associated with low pre-pregnancy BMI, including low-normal BMI (18.5–19.9 kg/m^2^). The dose-response relationship between the severity of maternal low pre-pregnancy BMI and LBW as well as SGA was represented in the low BMI range by RCS analysis. Although VLBW and ELBW were not statistically associated with maternal low pre-pregnancy BMI, a U-shaped relationship between pre-pregnancy BMI and VPTB and VLBW was observed in the RCS.

Our findings confirmed that the lower the pre-pregnancy BMI, the greater the risk of PTB, LBW, and SGA among Asian populations. Moreover, even low-normal pre-pregnancy BMI increased the risk of LBW and SGA. Therefore, in countries with a high prevalence of lean individuals, pre-pregnancy weight control in lean individuals of reproductive age can be an important intervention to prevent PTB, LBW, and SGA.

However, VPTB, VLBW, and ELBW were strongly associated with a higher pre-pregnancy BMI than lower BMI in our study. The present study analyzed these results based on maternal variables at the first prenatal visit and did not consider adverse complications during pregnancy. Therefore, VPTB, VLBW, and ELBW may be affected by adverse pregnancy complications because high pre-pregnancy BMI increases the risk of hypertensive disorders of pregnancy and gestational diabetes mellitus.

As in previous studies, the present study confirmed that maternal low pre-pregnancy BMI was an independent factor associated with PTB because a significant difference was identified after adjusting for confounding factors associated with adverse perinatal outcomes. Several studies have reported that maternal undernutrition, maternal infection, and inflammation due to lack of nutrients necessary for the immune system contribute to the relationship between low pre-pregnancy BMI and PTB [1, 9, 28]. However, it remains unclear why maternal low pre-pregnancy BMI increases the risk of PTB. Further research is needed to reveal the mechanism by which low pre-pregnancy BMI affects PTB.

The present study had several strengths. First, this was a large prospective cohort study that included pregnant Japanese individuals, with a high prevalence of thinness. Most previous studies on low pre-pregnancy BMI have been conducted in the United States and European countries, where the prevalence of thinness is low. Second, maternal pre-pregnancy BMI was categorized according to the appropriate BMI categories in the Asian population. In addition, the normal weight group was divided into two groups: low-normal weight and high-normal weight. Third, the RCS analysis was used to represent the non-linear relationship between maternal low pre-pregnancy BMI and adverse perinatal outcomes.

However, this study had several limitations. First, maternal gestational weight gain (GWG) was not included in the models because it was on the pathway from pre-pregnancy BMI to the outcomes. Future studies should note that the results of this study may not be representative of the general population due to the recent changes in the GWG guidelines. A meta-analysis on GWG [32] has reported that the optimal weight gain range was 14.0 kg to less than 16.0 kg for underweight individuals. However, this study has also reported that maternal pre-pregnancy BMI was more strongly associated with adverse maternal and infant outcomes than the amount of GWG. Also, dietary intake during pregnancy was not included in the models. Dietary intake during pregnancy, as well as GWG, may be related with PTB, LBW, and SGA. Second, any maternal complications that may have occurred during pregnancy were not considered as confounders because this study was conducted to predict the risk of adverse perinatal outcomes a pregnant individuals had at the time of her first visit. Third, a part of the maternal pre-pregnancy BMI was calculated using self-reported data when maternal height and pre-pregnancy weight were not available in medical record transcripts. Forth, the numbers of VPTB, VLBW, and ELBW were relatively small. Since our results demonstrated a trend toward increased risk of VPTB and VLBW with lower pre-pregnancy BMI, an increase in the number of cases may show a significant difference in adverse outcomes. Further research is needed to reveal the association between maternal low pre-pregnancy BMI and VPTB, VLBW, and ELBW. Fifth, this study may not apply to the non-Asian population as it was conducted on Japanese women.

## Conclusion

The present study demonstrated the association between the severity of maternal low pre-pregnancy BMI and adverse perinatal outcomes, including PTB, LBW, and SGA, in Japanese pregnant individuals. The dose-response relationship between the severity of low pre-pregnancy BMI and adverse perinatal outcomes was found in the low BMI range. Even low-normal pre-pregnancy BMI increased the risk of LBW and SGA. To reduce the risk of PTB, LBW, and SGA in lean individuals, it would be better to maintain a high-normal pre-pregnancy BMI. This study provides useful information for pre-conception counseling in lean individuals.

## Supplementary Information

**Additional file 1: Supplemental Table 1.** Odds ratios of maternal pre-pregnancy body mass index in the complete dataset (*n* = 84,366).

## Data Availability

Data are unsuitable for public deposition due to ethical restrictions and legal framework of Japan. It is prohibited by the Act on the Protection of Personal Information (Act No. 57 of 30 May 2003, amendment on 9 September 2015) to publicly deposit the data containing personal information. Ethical Guidelines for Medical and Health Research Involving Human Subjects enforced by the Japan Ministry of Education, Culture, Sports, Science and Technology and the Ministry of Health, Labour and Welfare also restricts the open sharing of the epidemiologic data. All inquiries about access to data should be sent to: jecs-en@nies.go.jp. The person responsible for handling enquiries sent to this e-mail address is Dr. Shoji F. Nakayama, JECS Programme Office, National Institute for Environmental Studies.

## Abbreviations

BMI: Body mass index
CI: Confidence interval
ELBW: Extremely low birth weight
GWG: Gestational weight gain
JECS: Japan Environment and Children’s Study
kNN: k-nearest neighbor
LBW: Low birth weight
OR: Odds ratio
PTB: Preterm birth
RCS: Restricted cubic spline
SGA: Small-for-gestational age
VLBW: Very low birth weight
VPTB: Very preterm birth
WHO: World Health Organization
