# Supplementary material for: Severity of low pre-pregnancy body mass index and perinatal outcomes: the Japan Environment and Children’s Study
Source: BMC Pregnancy Childbirth. 2022 Feb 11;22:121. doi: 10.1186/s12884-022-04418-3 (PMC8840606; doi:10.1186/s12884-022-04418-3)
Supplement: Supplementary file 1 — Additional file 1: Supplemental Table 1. Odds ratios of maternal pre-pregnancy body mass index in the complete dataset (n = 84,366). [file 12884_2022_4418_MOESM1_ESM.docx]

**Supplemental Table 1. Odds ratios of maternal pre-pregnancy body mass index in the complete dataset (n = 84,366)**

| Outcomes | Pre-pregnancy body mass index (kg/m^2^) | | | | | |
| --- | --- | --- | --- | --- | --- | --- |
|  | < 16.9 | 17.0-18.4 | 18.5-19.9 | 20.0-22.9 | 23.0-24.9 | 25.0 < |
| **PTB** |  |  |  |  |  |  |
| N (%) | 149 (4.00) | 519 (13.92) | 850 (22.79) | 1,291 (34.62) | 376 (10.08) | 544 (14.59) |
| Crude OR (95% CI) | 1.69 (1.42-2.02) | 1.22 (1.10-1.35) | 1.04 (0.96-1.14) | reference | 1.08 (0.96-1.21) | 1.53 (1.38-1.70) |
| Adjusted OR (95% CI) ^a^ | **1.74 (1.46-2.08)** | **1.25 (1.12-1.38)** | 1.07 (0.98-1.17) | reference | 1.01 (0.90-1.14) | **1.25 (1.13-1.40)** |
| **VPTB** |  |  |  |  |  |  |
| N (%) | 25 (3.45) | 84 (11.60) | 149 (20.58) | 249 (34.39) | 82 (11.33) | 135 (18.65) |
| Crude OR (95% CI) | 1.44 (0.95-2.17) | 1.01 (0.79-1.30) | 0.95 (0.77-1.16) | reference | 1.22 (0.95-1.57) | 1.95 (1.58-2.40) |
| Adjusted OR (95% CI) ^a^ | 1.46 (0.96-2.22) | 1.03 (0.80-1.32) | 0.97 (0.79-1.19) | reference | 1.12 (0.87-1.44) | **1.47 (1.18-1.84)** |
| **LBW** |  |  |  |  |  |  |
| N (%) | 357 (5.35) | 1,143 (17.13) | 1,712 (25.65) | 2,230 (33.41) | 581 (8.71) | 651 (9.75) |
| Crude OR (95% CI) | 2.52 (2.24-2.85) | 1.60 (1.49-1.73) | 1.23 (1.16-1.32) | reference | 0.96 (0.87-1.05) | 1.04 (0.95-1.14) |
| Adjusted OR (95% CI) ^a^ | **2.55 (2.26-2.88)** | **1.63 (1.51-1.76)** | **1.25 (1.17-1.34)** | reference | 0.92 (0.83-1.01) | **0.89 (0.81-0.97)** |
| **VLBW** |  |  |  |  |  |  |
| N (%) | 15 (3.75) | 48 (12.00) | 81 (20.25) | 131 (32.75) | 48 (12.00) | 77 (19.25) |
| Crude OR (95% CI) | 1.64 (0.96-2.80) | 1.10 (0.79-1.53) | 0.98 (0.74-1.29) | reference | 1.36 (0.97-1.89) | 2.10 (1.59-2.79) |
| Adjusted OR (95% CI) ^a^ | 1.71 (1.00-2.94) | 1.13 (0.81-1.59) | 1.00 (0.76-1.33) | reference | 1.24 (0.89-1.73) | **1.49 (1.10-2.01)** |
| **ELBW** |  |  |  |  |  |  |
| N (%) | 4 (2.76) | 14 (9.66) | 31 (21.38) | 56 (38.62) | 13 (8.97) | 27 (18.62) |
| Crude OR (95% CI) | 1.02 (0.37-2.81) | 0.75 (0.42-1.35) | 0.88 (0.56-1.36) | reference | 0.86 (0.47-1.57) | 1.72 (1.09-2.72) |
| Adjusted OR (95% CI) ^a^ | 1.04 (0.38-2.89) | 0.78 (0.43-1.39) | 0.90 (0.58-1.40) | reference | 0.78 (0.43-1.44) | 1.20 (0.73-1.95) |
| **SGA^b^** |  |  |  |  |  |  |
| N (%) | 350 (5.46) | 1,139 (17.78) | 1,719 (26.83) | 2,150 (33.56) | 547 (8.54) | 501 (7.82) |
| Crude OR (95% CI) | 2.56 (2.26-2.89) | 1.66 (1.54-1.79) | 1.29 (1.21-1.38) | reference | 0.94 (0.85-1.03) | 0.82 (0.74-0.91) |
| Adjusted OR (95% CI) ^a^ | **2.57 (2.27-2.90)** | **1.68 (1.56-1.81)** | **1.31 (1.22-1.40)** | reference | 0.91 (0.82-1.00) | **0.74 (0.67-0.82)** |

The adjusted odds ratios with statistical significance are presented in bold.

^a^The odds ratio compared to that of infants of mothers with the high-normal pre-pregnancy body mass index (20.0-22.9 kg/m^2^), adjusted for maternal age at delivery, parity (except for SGA analysis), assisted reproductive technology, maternal smoking status, maternal alcohol consumption, maternal educational background, history of preterm birth, medical history of hypertension, diabetes mellitus, autoimmune disease, and thyroid disease; PTB, preterm birth before 37 weeks’ gestation; VPTB, preterm birth before 34 weeks’ gestation; LBW, low birth weight (< 2,500 grams); VLBW, very low birth weight (< 1,500 grams); ELBW, extremely low birth weight (< 1,000 grams); CI, confidence interval. ^b^The total number of participants was 83,975.
